# Tongue metastasis as an initial manifestation of Distant metastasis in Oesophageal Adenocarcinoma

**Published:** 2014

**Authors:** Mutahir A. Tunio, Mushabbab AlAsiri, Muhammad Mohsin Fareed, Nagoud Mohamed Omar Ali

**Affiliations:** 1 Mutahir A. Tunio, MBBS, FCPS (Radiation Oncology), Assistant Consultant, Radiation Oncology, Comprehensive Cancer Center, King Fahad Medical City, Riyadh-59046, Saudi Arabia.; 2 Mushabbab AlAsiri, MBBS, FRSCI, MD, Consultant, Radiation Oncology, Comprehensive Cancer Center, King Fahad Medical City, Riyadh-59046, Saudi Arabia.; 3 Muhammad Mohsin Fareed, MBBS, FCPS (Radiation Oncology), Assistant Consultant, Radiation Oncology, Comprehensive Cancer Center, King Fahad Medical City, Riyadh-59046, Saudi Arabia.; 4 Nagoud Mohamed Omar Ali, MBBS, Anatomic Pathology, Comprehensive Cancer Center, King Fahad Medical City, Riyadh-59046, Saudi Arabia.

**Keywords:** Oesophageal adenocarcinoma, Metastasis, Tongue

## Abstract

Metastasis to the head and neck region from primary is rarest manifestation. Lung and breast carcinomas are the commonest malignancies to metastasize to the head and neck region. Oesophageal adenocarcinoma with metastasis to the oral cavity is a rarest presentation and is associated with dismal prognosis. Only few related case reports have been published so far. Her-in we report a case of 55 years old male who underwent radical oesophagectomy for adenocarcinoma of lower oesophagus twelve months back, now presented with hard mass in the right margin of tongue which was suspected as primary tongue carcinoma; subsequently was confirmed as metastatic oesophageal adenocarcinoma following excision. Two months after tongue excision, patient died of progressive metastatic disease.

## INTRODUCTION

Oesophageal cancer is the third most prevalent of all gastrointestinal malignancies worldwide and the second most common cancer in Saudi Arabia.^1^ Common histological variants are squamous cell carcinoma and adenocarcinoma. Both variants have similar clinical presentations despite their different locations. Oesophageal cancers commonly metastasize to liver, lungs and lymph nodes. Tongue is rare site of metastasis from oesophageal adenocarcinoma accounts for 0.1 to 0.2 per cent of all metastases.^2^ Tongue metastases typically remain asymptomatic, but could present as a painful hard masses and are considered as indicator of underlying high tumor burden and carry poor prognosis.^3^ Tongue metastasis of adenocarcinoma origin were first reported by Stanley et al in 1968 and since that only two more cases have been reported so far.^2^^-^^4^

In this report, we describe a patient with lower oesophageal adenocarcinoma who developed tongue metastasis, twelve months after neoadjuvant chemo radiation followed by oesophagectomy.

## CASE REPORT

**Fig.1:**
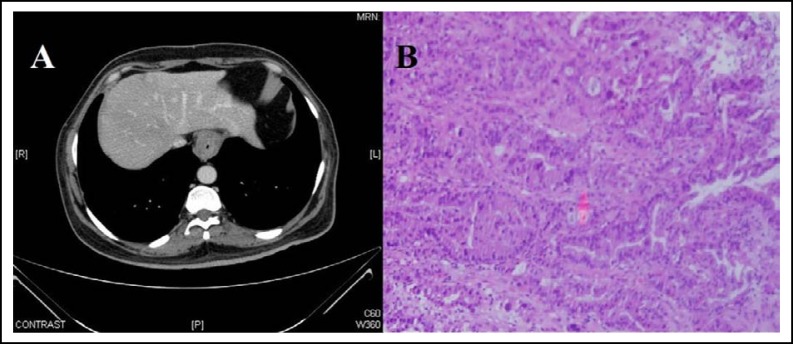
**(A)** Computed tomography (CT) of chest showing lower esophageal mass with para-oesophageal lymphadenopathy and **(B)** Esophagectomy specimen showing moderately differentiated adenocarcinoma

A 55 years old Saudi male presented in our clinic with history of rapidly progressing non-tender soft tissue mass in right lateral tongue since last 4 months. His past medical history revealed diabetes since last seven years controlled on metformin 500mg twice daily and family history was unremarkable. Past surgical history revealed that he underwent neoadjuvant chemoradiation 50.4 Gy in 28 fractions with concurrent oral capecitabine followed by radicaloesophagectomy for locally advanced lower oesophageal adenocarcinoma (radiological stage T3N1M0 and pathological stage ypT2N0M0) twelve months back in our hospital Fig.1 A and B.

On physical examination, he was severely mal-nourished with performance status ECOG-3, with mild pallor with no cyanosis and no palpable lymphadenopathy. A2x2 cm rounded, non-tender, without any ulceration soft tissue mass on right lateral tongue was noticed. The rest of clinical examination was unremarkable. Hematology, biochemistry, hepatic and renal function tests were found within normal limits.

**Fig.2:**
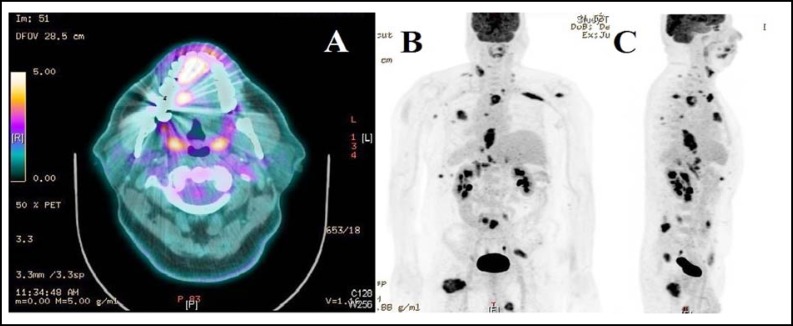
**(A) **18F-FDG PET-CTaxial image showing soft tissue mass in the right lateral border of tongue showing high maximum standardized uptake value (SUVmax 5.4), **(B)** Topogram showing hypermetabolic metastatic disease involving supraclavicular, mediastinal, retroperitoneal lymph nodes, multiple sites of muscle involvement (SUVmax 6.1).

**Fig.3:**
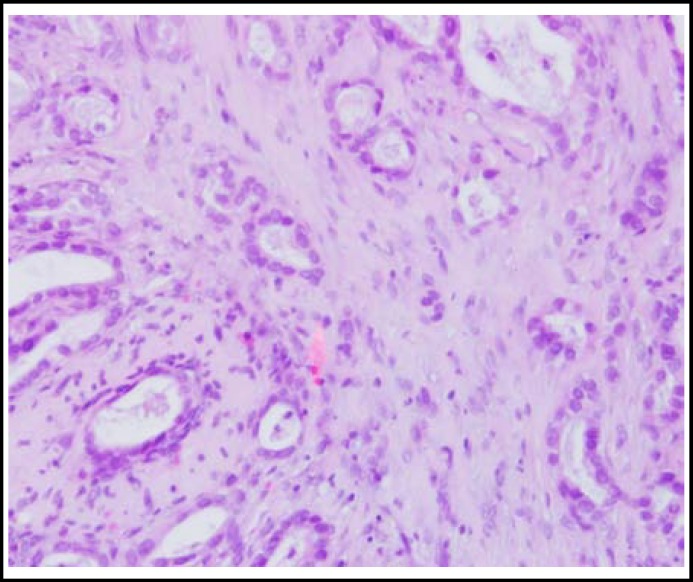
Hematoxylin and eosin staining showing the tongue with infiltrative nests of columnar cells (x100).

Computed tomography (CT) of head and neck region noted nodular focal thickening in the right posterior oral tongue without any cervical lymphadenopathy indicating primary tongue carcinoma or metastatic nodule. 18F-fluorodeoxyglucose positron emission tomography-computed tomography (18F-FDG PET-CT), showed soft tissue mass in the right lateral border of tongue showing high maximum standardized uptake value (SUVmax 5.4) consistent with second primary of tongue or metastasis Fig.2A. 18F-FDG PET-CT also revealed wide spread hypermetabolic metastatic disease involving supraclavicular, mediastinal, retroperitoneal lymph nodes, multiple sites of muscle involvement (both shoulders, right pectoralis, chest, abdominal walls, paraspinal and thigh) (SUVmax 6.1) Fig.2B. Excisional biopsy was taken from right tongue lesion. Histopathology showed glandular nests within muscular layer and immunohistochemical examination showed CK7, CKX2 positivity and negativity for PSA and Her 2-neu and confirmed the diagnosis of metastatic adenocarcinoma of oesophagus Fig.3.

After discussing, the case in multi-disciplinary board, patient was started on palliative duplet chemotherapy based on cisplatin and irinotecan. After one cycle of chemotherapy, patient died with progression of disease on day 60 of initial presentation as tongue lesion.

## DISCUSSION

Tongue metastases from oesophageal adenocarcinoma are extremely rare. In past decades, patients with adenocarcinoma of the esophagus used to die from local failures, however because of improved locoregional control due to advent of tri-modality treatments, such as neoadjuvant chemoradiation followed by oesophagectomy and novel chemotherapeutic agents, in most patients overall survival has been increased and recurrences seen have mainly been from hematogenous spread, as seen in our patient.^5^

Metastasis to the head and neck region from other primary malignancies is rarest. Renal cell carcinoma, lung, breast carcinomas are the commonest malignancies to metastasize to the head and neck region.^6^ The common presenting symptoms of metastatic deposits in the head and neck region are; enlarged solitary mass, epistaxis, nasal obstruction and lymphadenopathy, depending on the location and the extent of invasion by metastatic deposits.^7^

The possible routes for tongue metastasis suggested are the hematogenous and lymphatic circulation. The presences of valve-less interconnecting vertebral veins allow metastases to bypass the heart and lungs and communicate directly with the veins of the head and neck region. The vertebral veins in turn communicate superiorly with the pterygoid plexus, cavernous sinus and superior portion of the pharyngeal plexus, hence providing a pathway for metastasis to the tongue.^8^ Head and neck metastases are commonly associated with widespread metastasis of other sites as seen in our patient with dismal prognosis.

A panel for immunohistochemical staining is confirmatory for the diagnosis of tongue metastasis and to differentiate from primary tongue carcinoma, however 18F-FDG PET-CT can be important imaging modality to reach at the diagnosis as the SUVmax has been shown to correlate with tumor differentiation and metastases in esophageal cancer.^9^ Previous three case reports have suggested that patients with tongue metastasis from oesophageal adenocarcinoma have expected 5 year survival is less than 10%, therefore all treatment options for tongue metastasis are with palliative intent.

In conclusion, tongue metastases from oesophageal cancers are rare and carry poor prognosis because most of these patients have widespread underlying metastatic disease. Clinical and histologic differentiation between metastatic and primary tongue carcinomas is very important as both have different treatment options.

## Authors contribution:

*Mushabbab AlAsiri:* Concept of study and Manuscript editing.

*Mutahir Ali Tunio, Muhammad Mohsin Fareed:* Manuscript writing.

*Nagoud Mohamed Omar Ali:* Pathological assistance.

All authors approved the final manuscript for publication.
